# MED19/390: Developing a Digital Textbook on Biomedical Technology for Health-Care Professionals

**DOI:** 10.2196/jmir.1.suppl1.e62

**Published:** 1999-09-19

**Authors:** B Spyropoulos

**Affiliations:** ^1^Technological Educational Institution of AthensAthensGreece

**Keywords:** Digital Textbook, Teaching Materials, Biomedical Technology

## Abstract

**Introduction:**

The main objective of the developed Digital Textbook is to present the Technology associated to the most important Diagnostic and Treatment Procedures, applied in contemporary Biomedical Practice. The target-group of this on-line volume comprise of both, students and those who are already engaged in professional work, such as physicians, nurses, engineers, physicists and other people trained in health-care related activities.

**Methods:**

The educational method used includes the presentation of the physical principles, upon which the procedures and equipment under investigation are based, as well as, the most common technical implementation of them. Furthermore, care has be taken to relate the described devices to the corresponding Hospital Departments or Health-Care Facilities, and some simple demonstrations and quizzes are included, that give the user the opportunity to become a little bit more familiar with the clinical hardware. For the development of the digital textbook, HTML was used, so that it can be used on any platform. The content of the presentation is a combination of theoretical knowledge and practice oriented information. Because of the enormous extend of the subject matter, the structure of the text, is rather that of an overview. The languages used are Greek and English.

**Results:**

The developed system consists of educational modules, which presently include topics on the following subjects:

- In vivo Diagnostics Technology.
- In vitro Diagnostics Technology.
- Medical Imaging Technology.
- Operating Room and Intensive Care Technology.
- Radiation Treatment Technology.
- Environmental Protection in the Modern Hospital.
- Hospital Organization and Biomedical Technology Management.

**Discussion:**

The textbook covers educational needs concerning the physical principles, the most common implementations of the medical equipment and the management of the infra-structure of the basic units of the modern Hospital. The new electronic media may offer a cost-effective "digital alternative", for individuals and Institutions. An appropriate combination of traditional and on-line educational activities, could contribute to the promotion of interdisciplinary research and to the improving of the interaction between senior and junior colleagues, in the training procedure.

